# Experimental Study on the Flexural Creep Behaviors of Pultruded Unidirectional Carbon/Glass Fiber-Reinforced Hybrid Bars

**DOI:** 10.3390/ma13040976

**Published:** 2020-02-21

**Authors:** Hiran Mayookh Lal, Guijun Xian, Sabu Thomas, Lei Zhang, Zhonghui Zhang, Huili Wang

**Affiliations:** 1 School of Civil Engineering, Harbin Institute of Technology, Harbin 150090, China; hiran009@yahoo.co.in; 2 International and Inter University Centre for Nanoscience and Nanotechnology (IIUCNN), Mahatma Gandhi University, Kerala 686560, India; sabuthomas@mgu.ac.in; 3 Shengli Oilfield Company, China Petroleum & Chemical Corporation (SINOPEC), Dongying 257100, China; zhanglei976.slyt@sinopec.com (L.Z.); zhangzhonghui.slyt@sinopec.com (Z.Z.); wanghuili.slyt@sinopec.com (H.W.)

**Keywords:** hybrid FRP bar, creep behavior, freeze–thaw cycles, creep deflection, viscoelastic behavior

## Abstract

Unidirectional pultruded glass/carbon hybrid fiber-reinforced polymer (HFRP) bars with a diameter of 19 mm have recently been developed for various structural applications. In this study, the creep behavior of HFRP bars caused by bending was experimentally evaluated under different conditions. Our creep study included freeze–thaw preconditioned and unconditioned HFRP bars. The rate of strain and deflection were monitored continuously for a duration of 5000 h. The bars were further tested for creep under the combined effects of mechanical loading and induced thermal cycles, while continuously monitoring the strain rate. Stress levels of 50% to 70% were selected for our creep study. The creep behavior of the bars was analyzed utilizing Findley’s power-law model. On the basis of the linear approximation of Findley’s power law, modulus reductions of approximately 21%, 19%, and 10.75% were calculated for combined freeze–thaw/creep-loaded, freeze–thaw pretreated, and unconditioned HFRP bars, respectively, over a service period of 50 y. The time-dependent deflection of HFRP bars was analyzed by coupling Findley’s power-law model with Euler Bernoulli’s beam theory. The creep deflection intensified by 26.6% and 11.1% for preconditioned and untreated bars, respectively, after a service period of 50 y. The microstructures of HFRP bars was also examined utilizing scanning electron microscopy.

## 1. Introduction

Pultruded fiber-reinforced polymers (FRPs) have achieved widespread acceptance in civil engineering and structural applications as a competitive alternative to conventional structural materials, such as steel [1,2,3]. The main advantages of FRPs are low thermal expansion, light weight, excellent corrosion resistance, high strength-to-weight ratios, and excellent fatigue resistance, particularly for carbon–FRP composites (CFRPs) compared with steel [4]. However, FRPs also have limitations in terms of anisotropic behavior, creep rupture, poor fire resistance, weak transverse shear strength, and being brittle in nature, with linear elastic behavior up to rupture [5,6].

FRP bars/rods are widely used as structural materials in civil engineering, such as for external and internal tendons in reinforced concrete beams and bridge decks [7,8,9,10]. For particular cases, such as bent FRP bars in external pre-stressed tendons, and conventional concrete beams with unbonded/bonded harped FRP internal reinforced tendons, which usually account for stress concentrations [6,11,12,13], the formation of additional stresses based on bending increases the risk of creep and creep rupture. This risk increases as the bending angle of FRP tendons increases [11]. FRP bars as anisotropic materials provide lower transverse strength and modulus compared with their longitudinal strength and modulus [6]. As another point of consideration, FRP bar/rod manufacturing companies prefer to make long continuous bars/rods based on economic and efficiency concerns. Fabricating long bars/rods reduces the number of connections between pieces, which can prevent coupling failures [14]. Continuous bars/rods are generally stored in reel holders after manufacturing, where reel curvature depends on the diameter and length of the manufactured bars/rods. The long-term storage of FRP bars/rods in reels induces additional bending stress and increases the risk of permanent deformation (creep) based on the viscoelastic nature of FRPs, which is attributed to the delayed viscous part of the polymer matrix [15]. Overall, the low transverse flexural strength of FRP composites is regarded as a critical issue and the adverse effects of creep caused by bending must be considered before applying FRP bars as an engineering material.

Recently, the application of FRP bars has been extended to submarine structures, oceanographic profilers, and subsea oil industry applications, where FRP load-bearing bars/rods are utilized to replace steel members. When considering FRPs as structural elements, they are not only subjected to structural loads, but also varying temperatures, moisture, and other crucial factors that influence the long-term behavior of FRP composites [16]. FRP composites become fragile under both extremely high and low temperatures, which strongly influences their mechanical behavior [17,18,19]. The effects of these environmental factors must also be considered for the long-term outdoor storage of continuous FRP bars in reel holders or coiled storage systems. These factors must be considered by designers and manufacturers to understand how the behavior of FRPs affects the long-term design life of structures. Among FRP composites, glass–FRP (GFRP) composites are widely used for various structural applications owing to their cost efficiency [20,21]. However, GFRPs possess poor mechanical properties and low durability, which limits the scope for their application [22]. In contrast, CFRPs are widely used owing to their superior mechanical properties (stiffness and strength) and excellent fatigue and corrosion resistance. However, CFRPs are limited by poor cost efficiency [23,24]. The hybridization of GFRPs and CFRPs in a single matrix has recently emerged as a technique to leverage the advantages of both types of composites for various structural applications. For example, novel carbon/glass hybrid FRP (HFRP) composite bars/rods with core/shell structures have been developed [25,26,27].

To date, few investigations have analyzed the long-term viscoelastic creep behavior of HFRP composites or the effects of freeze–thaw cycling on the creep of HFRPs. However, several investigations have focused on the long-term behavior of the material durability of FRP/HFRP under the combined effects of sustained loading under weathering conditions [28,29]. Wei et al. [28] studied the freeze–thaw resistance of carbon, basalt, and glass fiber HFRP sheets under sustained tensile loading for samples subjected to 300 freeze–thaw cycles. The HFRP and basalt FRP sheets exhibited better freeze–thaw resistance than the CFRP and GFRP sheets. The HFRP sheets exhibited the most stable tensile properties. The degradation of FRP sheets is mainly attributed to sustained loads, which accelerate the rate of moisture propagation in the resin matrix, thereby degrading material properties in freeze–thaw environments. Wu et al. [29] investigated an FRP bridge deck material under a sustained load for 10,000 h of freeze–thaw cycle conditioning. On the basis of a mechanics model, they analyzed the effects of sustained flexural loading and environmental exposure on the degradation of flexural strength and stiffness. The effects were greater for composites subjected to sustained loading after freeze–thaw cycle conditioning. The samples that were not loaded exhibited an insignificant decrease in properties. The thermal incompatibility owing to the difference in coefficient of thermal expansion (CTE) between the constituent materials of FRP composites affects the material property when FRP composites are exposed to an freeze-thaw (FT)environment. In general, the resin matrix in FRP composites have CTEs that are at least an order of magnitude greater than those of the fibers. At low temperature, residual stresses are developed at the resin matrix–fiber interface owing to the differences in CTE between the two materials. In the FT environment, the cyclic stresses are repeated at the resin matrix–fiber interface, resulting in damage and poor service life [28,30]. The behavior of a hybrid fiber system is more complex than that of a homogenous fiber system in moist environments [31]. The creep behavior of HFRPs with special carbon fiber cores and glass fiber shells based on bending under the effects of cyclic thermal conditioning and mechanical loading requires further research.

Several studies have addressed the creep behavior of pultruded unidirectional FRP profiles [32,33,34,35,36]. We aimed to investigate the creep behavior of unique carbon fiber core/glass fiber shell HFRP bars under bending in various weather conditions. The effects of creep on untreated samples, freeze–thaw pretreated water-soaked samples, and combined mechanically loaded samples under freeze–thaw cycling conditions were addressed in this study. Moderate to high stress levels (ranging from 50% to 70% of ultimate strength) were considered to evaluate the creep behavior of large-diameter HFRP bars, to meet the requirements of the design and maximum applicability of such type of novel FRP composite bar as a structural element.

## 2. Materials and Methods

### 2.1. HFRP Bars

**Figure 1:**
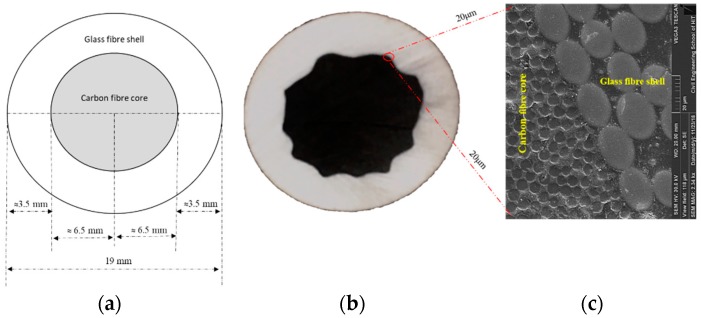
Hybrid fiber-reinforced polymer (HFRP) bar cross-sectional view: (**a**) Diagram representing the cross-sectional dimensions of HFRP bars; (**b**) Cross section of hybrid HFRP bars; (**c**) Enlarged scanning electron microscopic image showing the interface between glass fiber shell/carbon fiber core.

The HFRP bars considered in this study have a circular cross section containing two sections: a skin layer of GFRP and core layer of CFRP. The overall diameter of an HFRP bar is 19 mm. The thickness of the skin layer is approximately 3.5 mm and the radius of the inner core layer is 6.5 mm. In our experimental program, the test span for the HFRP bars was 900 mm for all types of tests. Figure 1 presents a cross-sectional view of the HFRP bar utilized in our tests. The bars are manufactured utilizing the pultrusion process and the resin matrix is a bisphenol-A epoxy resin (AirstoneTM 1122E, Blue Cube Chemicals Company, Zhangjiagang, China). The volume fractions of glass fibers and carbon fibers are approximately 70%. The used glass fiber is a common E-GF produced by Taishan Glass Fiber Co. (Taian, China) with tensile strength of 2.1 GPa and modulus of 72 GPa. Carbon fiber is 12 k produced by Sinopec. Co. (Shanghai, China) with the tensile strength of 3.5 GPa and modulus of 230 GPa.

### 2.2. Freeze Thaw Conditining

The HFRP bars were subjected to freezing and thawing cycles inside an environment chamber (ZHS-30 Multifunctional Climate Laboratory, designed and built by the School of Civil Engineering, Harbin, China). Each cycle lasted 12 h with 5 h of continuous freezing and 5 h of continuous heating and 2 h of transitional time between each cycle. The temperature changed from −20 to 20 °C during each cycle with a heating/cooling rate of 0.67 °C/min. Freeze–thaw conditioning of HFRP bars was performed in two different ways in this study. The first involved a pretreatment cycle in which specimens were soaked in tap water above 0 °C for 160 thermal freeze–thaw cycles. A total of 11 HFRP bar specimens were subjected to freeze–thaw pretreatment cycles. The samples were then removed from the environment chamber and subjected to time-independent short-term property tests and time-dependent long-term creep analysis within a span of 48 h. For the second type of conditioning, the specimens were subjected to combined thermal freeze–thaw cycles with mechanical creep loading for 120 thermal cycles with relative humidity maintained at approximately 75%. A total of six HFRP bar specimens were used for combined thermal freeze–thaw cycles with mechanical creep loading. The temperature and humidity outline was displayed on the computer interface provided with the chamber.

### 2.3. Time-Independent Static Flexural Test

**Figure 2:**
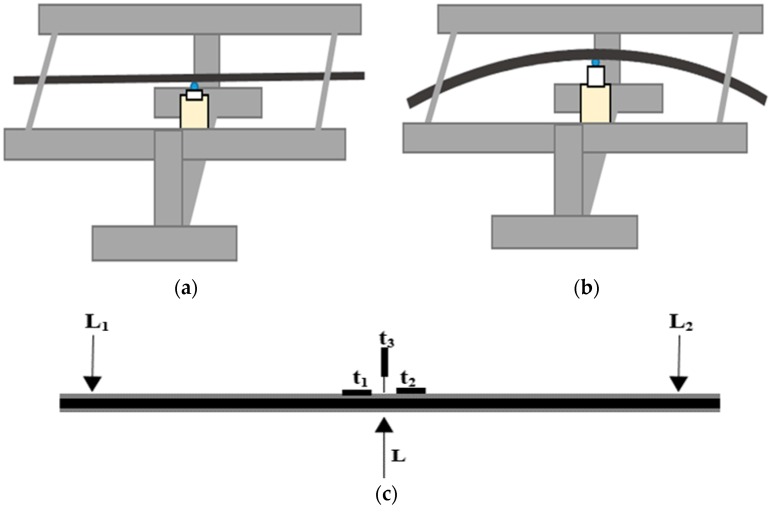

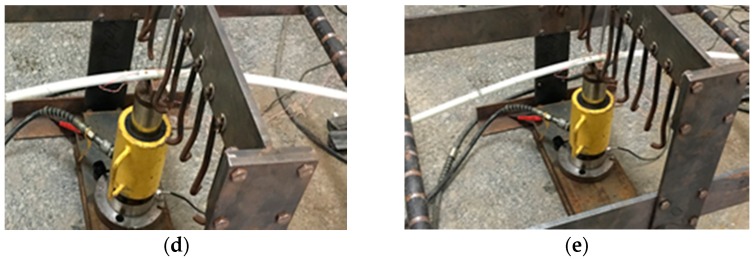
Short-term test fixture setup. (**a**) Diagram showing HFRP bars before bending; (**b**) Diagram showing HFRP bars on bending with an upstroke of jack shaft; (**c**) Location of strain gauges (t_1_ and t_2_), linear variable differential transducer (LVDT) (t_3_), supports at both ends (L_1_ and L_2_), and applied load on mid-span (L); (**d**) and (**e**) photographs of the test setup.

The flexural behavior of the HFRP bars was tested under three-point bending to identify ultimate strength and deflection values at room temperature. These tests were carried out for both the pre-conditioned freeze–thaw samples soaked in water above 0 °C with 0% load (FT samples) and the untreated samples (RT samples). A total of five HFRP bar specimens were tested for both FT samples and RT samples to obtain average time-independent static flexural parameters. The test was conducted at room temperature (20 °C) with relative humidity at approximately 66%. The fixture utilized for time-independent testing was custom designed and manufactured by the Laboratory of FRP Composites and Structures at the Harbin Institute of Technology, Harbin, China. The diagrams and photographs of the static bending test fixture are presented in Figure 2. The steel bars on each side of the fixture can support the ends of HFRP bars. The distance between the supporting steel bars is the test span of the HFRP bars. The plates that hold the supporting steel bars are connected to the base legs, which are connected to a fixed base beam. A hydraulic jack (RSC 10150, Zupper, Zhejiang, China) with a stroke of 150 mm and maximum capacity of 10 T is installed on the base beam. The upstroke of the piston can induce mid-span bending of the HFRP bar test specimens and loading is performed manually, resulting in an average load control rate of approximately 0.02 kN/s. Strain gauges are installed along the axial surface of the bar to measure average axial strain during bending. A linear variable differential transducer (LVDT, SOP, Jiangxi, China) is utilized to measure the bending deflection corresponding to the applied load. Figure 2c presents a schematic of a sample cross section of an HFRP bar and the corresponding arrangement of strain gauges, as well as the LVDT utilized for monitoring bending strain and deflection. Time-independent flexural testing was performed to determine the ultimate stress $\sigma_{xl}$, ultimate strain $\varepsilon_{x0}$, ultimate bending load  fl, and ultimate deflection $\delta_{fl}$ of the HFRP bars.

### 2.4. Time-Dependent Creep Test at Room Temperature

**Figure 3:**
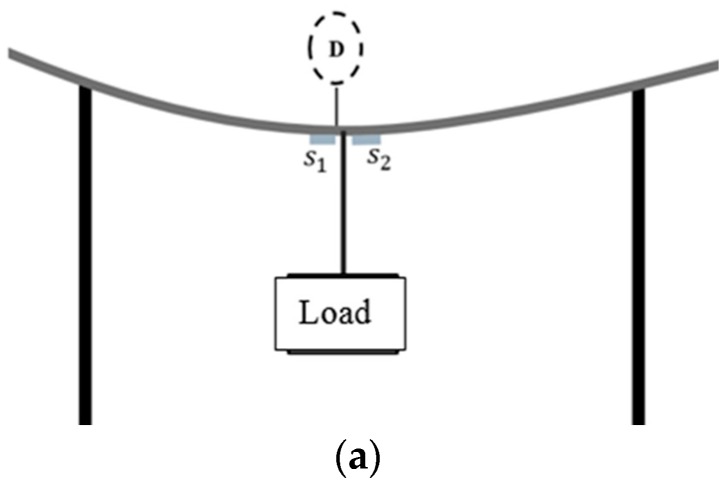

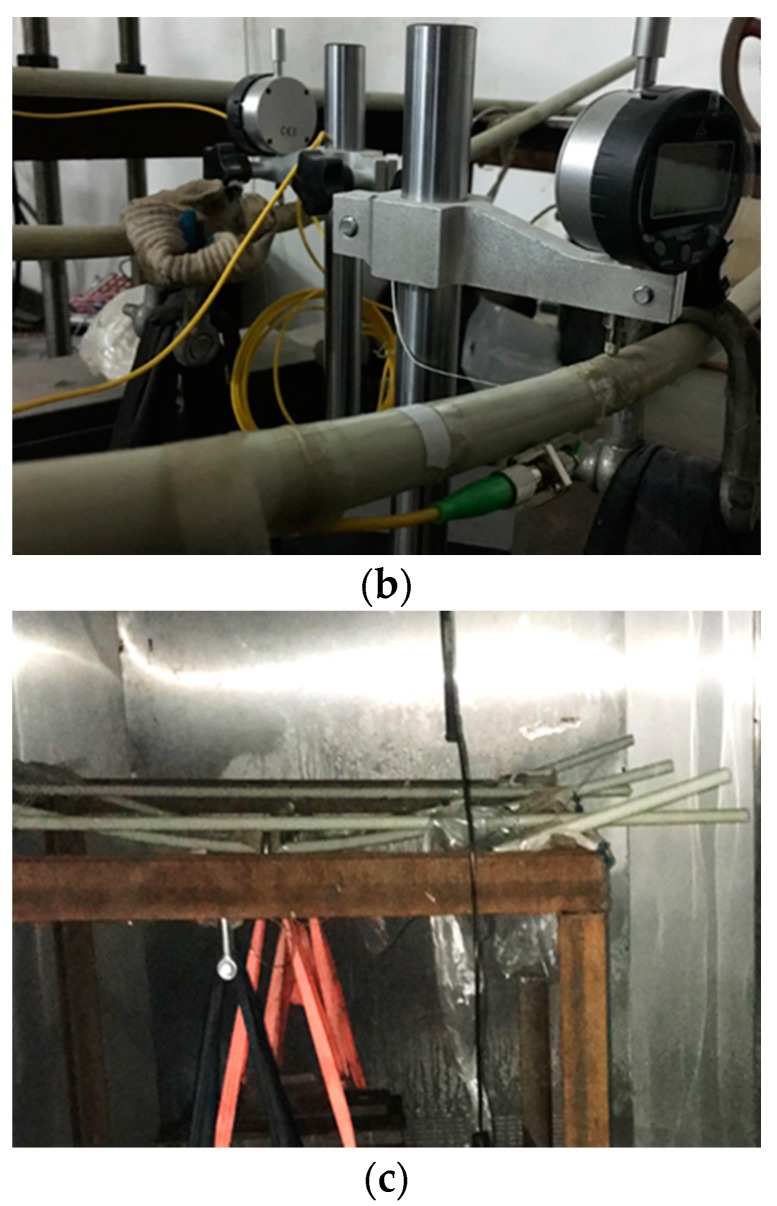
Creep test setup: (**a**) Diagram of creep test setup showing the position of dial gauge D and the location of strain sensors $\;(S_{1}$ and $S_{2})$; (**b**) Creep test in laboratory condition; (**c**) Creep test inside the environmental chamber.

In our flexural creep experiments, FT and RT samples were subjected to sustained loading at room temperature inside the laboratory. The samples were placed in a three-point bending configuration by suspending heavy metal blocks at their mid-span points to achieve stress levels corresponding to 50%, 60%, and 70% of the ultimate stress $\sigma_{xl}$. The loads were fixed according to the corresponding stress and deflection values based on the static flexural tests described earlier. For each stress level, three HFRP bars were tested for creep to extract accurate and average rate of change of strain. The creep tests at room temperature were conducted for 5000 h. The creep fixture is designed with the capability to support three samples with the suspension of dead loads. Two parallel steel frames support the samples. A diagram and photographs of the creep test fixture are presented in Figure 3. The distance between the supporting frames is the test span (900 mm) of the HFRP bars. The supporting frames are connected to four transverse legs and two additional supporting legs, which are welded to the base of the fixture. The creep tests for the RT and FT samples were conducted in laboratory at the Harbin Institute of Technology, Harbin, China at temperatures ranging from 16 to 20 °C. Time-dependent creep strain and deflection were monitored continuously throughout the creep study. The strain rate was manipulated by utilizing fiber Bragg grating (FBG) strain sensors (Shenzhen Taichengguang, Ltd., Shenzhen, China), which were fixed to the bottom surfaces (tension zone) of all specimens using epoxy adhesive. When surface-mounted FBGs are stretched, the grating period generates changes in the wavelength of reflected UV light inside the core fibers of the sensors. These changes in wavelength are then converted into strain rates [37,38]. The rate of change of the grating period was monitored continuously and the values of the wavelength outputs were displayed on a monitor connected to the sensors, and then converted into micro-strain values. Creep deflection measurements were obtained from a digital displacement transducer (stroke between 12.5 and 30 mm with 0.001 mm precision). Figure 3b presents the arrangements of strain sensors and dial-gauges utilized for the creep study at room temperature. Dummy FBGs were also placed close to the mechanically loaded samples to prevent temperature variations from interfering with strain monitoring.

### 2.5. Time-Dependent Creep Test under Combined Freeze–thaw Cycle Conditioning and Loading

The viscoelastic behavior of the HFRP bars was further characterized by monitoring their deformation under the combined effects of mechanical loading and thermal freeze–thaw cycle conditioning. The stress levels for this creep study were 50%, 60%, and 70% of the ultimate stress $\sigma_{xl}$ based on the static bending tests of the RT samples. The HFRP bars tested for each stress level is limited to two sample considering the limitation of space inside the environmental chamber for occupying more samples as well as the long duration of the creep test. The creep test for HFRP bars under combined loading and freeze–thaw cycle conditioning was conducted inside the environmental chamber, as shown in Figure 3c, with 120 thermal cycles between −20 and 20 °C. For the FT-L samples, creep strain was monitored utilizing FBGs mounted on their bottom surfaces (tension zones) throughout the creep tests. The outputs of viscoelastic strain may influence the temperature components coupled with displacement components. In our experimental program, strain rate measurement was achieved by applying a temperature compensation method by placing an identical FBG with zero mechanical strain near the active strain-measuring FBGs. The final deformation values are the average values of two FBG strain sensors and two dummy FBGs for compensation. In Figure 3c, it can be seen that the arrangement of dummy FBGs glued to the HFRP bar surfaces with zero mechanical strain is placed very close to the mechanically loaded HFRP bar samples. Deflection creep was not analyzed for the FT-L samples.

### 2.6. Scanning Electron Microscopy

The interface cross sections of the HFRP bars were analyzed morphologically using scanning electron microscopy (SEM, Vega3, Tescan, Brno, Czech Republic). Sample preparation involved cutting cross sections of the HFRP bars with a thickness of 5 mm. The resulting specimens were then coated with epoxy resin and polished thoroughly. The samples were mounted on aluminum plates with the scanning surfaces of the HFRP bar cross sections pointed upward. A thin layer of gold (7 ± 2 nm) was deposited as a conducting material utilizing an E5200 Auto Sputter Coater, Cambridge, UK. prior to testing.

## 3. Results and Discussion

### 3.1. Short-Term Flexural Properties

The data acquired from the short-term time-independent flexural studies (for 900 mm spans) of RT and FT samples are listed in Table 1. The theoretical results were obtained from estimations based on simple beam calculations. The initial flexural modulus $E_{x0}$ in this experiment was determined by following the ASTM D 4476-09 [39] standard. $E_{x0}$ was assumed to be identical for all samples.

**Figure 4:**
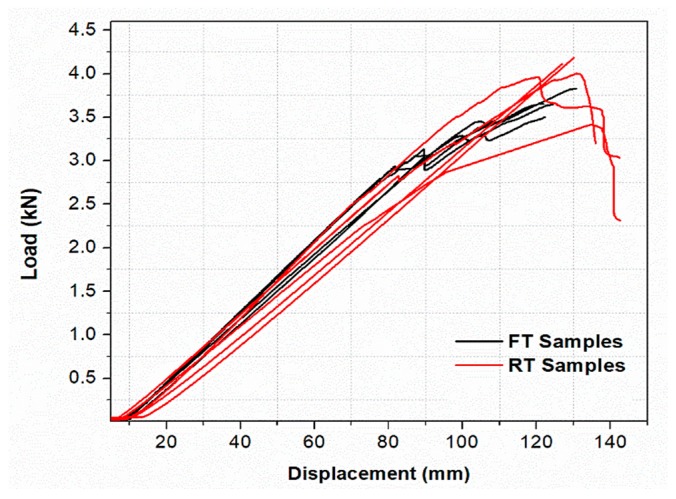
The short-term test results of RT samples and FT samples.

A load–displacement curve that represents test result of all samples tested for the static flexural test is plotted in Figure 4. The samples were subjected to preconditioning freeze–thaw cycles to induce depletion on the mean values of their static properties ($\sigma_{xl}$, $\varepsilon_{x0}$, and $\delta_{fl}$ were reduced by the average of 11%, 14%, and 8%, respectively). The reduced static parameters were considered for the creep study of the FT samples. Each stress level of the FT samples was approximately 11% lower than the corresponding stress level of the RT samples. The average results of derived stress levels and its corresponding strain values used for the creep study are given in Table 2.

**Figure 5:**
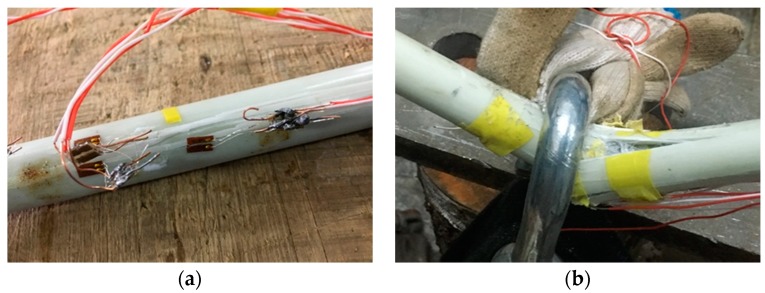
Bending failure modes: (**a**) Failure occurred during static flexural test; (**b**) Failure of HFRP bars occurred as a result of suspending dead loads up to its maximum capacity.

During the short-term experiment, the failure of HFRP bars occurs following the delamination of glass fiber shell in the tension zone in combination with cracking on the tension surface, as shown in Figure 5a. The material then exhibits nonlinear behavior. The static flexural tests were conducted to determine $\sigma_{xl}$, $\varepsilon_{xl}$, and $\delta_{fl}$. These values are derived from the point at which the material loses its integrity under the applied load. This point was considered to represent the ultimate load $f_{l}$ and ultimate stress $\sigma_{xl}$ for the HFRP bars in this study.

To compare failure modes, the HFRP bars were loaded to the point of final fiber breakage by suspending steel blocks from the bars (steel blocks with known weights were selected prior to our experiments). We monitored strain and deflection values for calculations and comparisons. Failure of the HFRP bars occurred instantaneously following longitudinal separation of the glass fiber shells. As the applied load increased above a certain limit, a nonlinear shift in deformation accompanied by shell delamination and failure of the carbon fiber core eventually occurred, resulting in the failure of the HFRP bars, as shown in Figure 5b. A comparison of the failure modes for both types of static bending tests of HFRP bars is presented in Figure 5.

### 3.2. Long-Term Creep Testing

**Figure 6:**
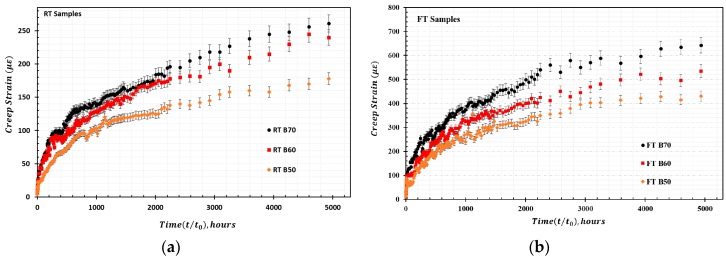
HFRP creep strain for samples loaded at 50–70% ultimate stress: (**a**) Creep strain for untreated RT samples; (**b**) Creep strain for freeze–thaw pretreated FT samples.

**Figure 7:**
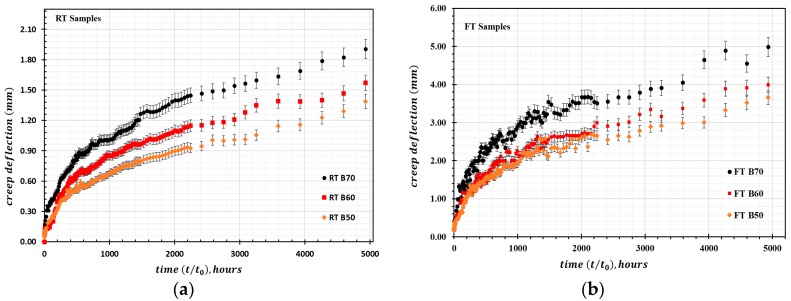
HFRP creep deflection for samples loaded at 50–70% ultimate stress: (**a**) Creep deflection for untreated RT samples; (**b**) Creep deflection for freeze–thaw pretreated FT samples.

The RT samples and FT samples were tested for creep at room temperature inside our laboratory to determine the average creep strain for each type of stress variation in the RT and FT samples as a function of time. The derived creep curve for FT and RT samples is presented in Figure 6. The evaluation of mid-span deflection at each stress level for the RT and FT samples as a function of time is presented in Figure 7. It should be noted that the creep strain and deflection for the FT samples at each stress level are greater than the corresponding values for the RT samples, meaning the percentage increase in creep is greater for the FT samples. The samples only exhibited primary (instantaneous) creep and secondary (steady) creep behaviors during this creep study. Secondary creep existed for a long duration throughout the creep study, but tertiary creep behavior was absent. This behavior is attributed to the typical viscoelastic nature of polymer matrix composites. As expected, the overall creep strain and deflection increase with higher stress levels. The percentage increase in creep strain $\varepsilon_{xt}\;\left(\%\right)$ is greater for the FT samples compared with that for the RT samples. For the RT samples, the maximum $\varepsilon_{xt\;}\left(\%\right)$ value for the duration of t = 5000 h for the load levels of RT B50 and RT B70 are approximately 2.6% and 3.2%, respectively. In contrast, the $\varepsilon_{xt}\;\left(\%\right)$ values for FT B50 and FT B70 at t = 5000 h are approximately 7.21% and 7.78%, respectively. It is also worth noting that the empirically derived loads corresponding to the stress levels for the RT and FT samples during the creep tests are not the same. The divergence for selected stress levels for the RT samples is greater than 11% relative to the FT samples.

**Figure 8:**
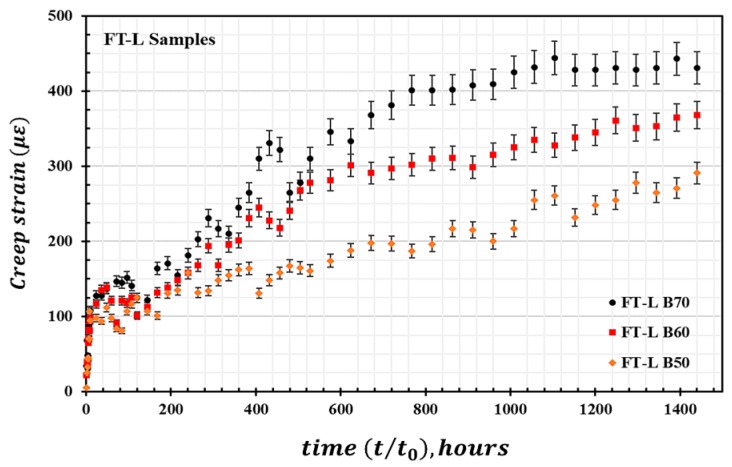
HFRP creep strain for samples loaded at 50–70% ultimate stress for FT-L samples.

Figure 8 presents the derived average creep strain of the FT-L samples tested inside the environmental chamber under the combined effects of induced thermal cycles and mechanical loads. The percentage increase in creep strain $\varepsilon_{xt}\;\left(\%\right)$ during 120 thermal cycles for the load levels of FT-L B50 and FT-L B70 are approximately 4.12% and 4.60%, respectively. When comparing the amplitudes of creep in Figure 6 and Figure 8, the primary creep stage or instantaneous increase in creep strain is notably higher for the FT-L samples compared with those for the FT and RT samples. In the secondary creep region, the FT samples exhibit a percentage increase in creep strain relative to the FT-L and RT samples. On comparing the creep amplitudes between FT B70 and FT-L B70 samples at a reference time of 1400 h, it is found that the creep amplitude of FT samples is approximately 5.10% higher than FT-L samples. A higher creep effect in FT samples is expected because of the higher moisture uptake/matrix softening during pretreatment [40] (assuming that the drying effects are limited during the test procedure). This can also be attributed to the longer duration for which the FT samples were subjected to sustained loads compared with the FT-L samples.

Dissimilarities in elastic responses in the primary creep region for different materials may be related to the development of internal stress in the resin matrix/fiber interface during freeze–thaw treatment. The different thermal expansion coefficient between the carbon fiber and resin matrix could contribute to the development of additional internal stress during freeze–thaw treatment [41]. The mismatching coefficients of thermal expansion (CTEs) between the resin matrix, glass fiber shell, and carbon fiber core inside the HFRP bars could also contribute to the development of micro-defects based on the presence of residual stress in the matrix and particularly at the core/shell interface of HFRP bars during the freeze–thaw treatment. On the basis of this dissimilarity in the behaviors of HFRP bars under different environmental conditions [28,42], we can conclude that the creep behaviors of conventional FRPs are significantly different from those of HFRP systems under thermal cycling conditions based on the aforementioned factors.

### 3.3. Evaluation of Creep and Viscoelastic Modelling Utilizing Findley’s Power Law

Owing to its simplicity, Findley’s power law is the most commonly applied mathematical model for analyzing the long-term creep behavior of reinforced composite materials under constant stress [32,33,34,36,43,44,45,46]. The creep behavior of any FRP material under constant stress can be expressed as

(1) $$\varepsilon_{xt}=\varepsilon_{x0}+m{\left(\frac{t}{t_{0}}\right)}^{n}\;$$

Equation (1) was utilized to predict the axial creep strain under flexural loading in this study, where $\varepsilon_{xt}$ is the time-dependent creep strain, $\varepsilon_{x0}$ is the stress-dependent initial elastic deformation, m  is the stress-dependent creep coefficient for axial strain, and n is the stress-independent material constant. m and n can be expressed as creep amplitude and time exponent, respectively. t  is the time in hours and $t_{0}$ is the unit time (1 h). The values of constants can be extracted from the experimental creep strain by rearranging Findley’s power law equation by taking the log of both sides.

**Figure 9:**
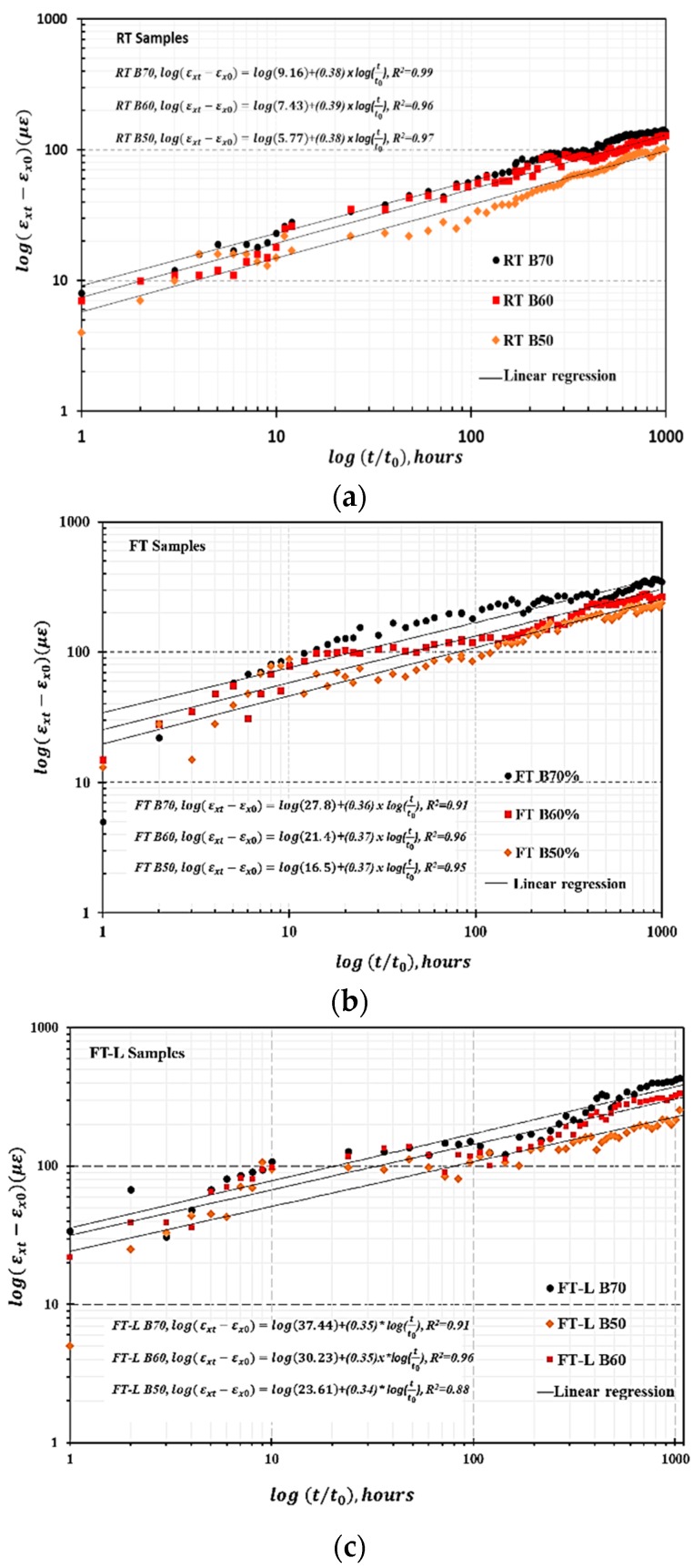
Evaluation of creep parameter *n* and *m* (**a**) for RT samples, (**b**) for FT samples, and (**c**) for FT-L samples.

The relationship between experimental creep strain data and time is plotted logarithmically in Figure 9a,c. The linear dependence between $\log\;(\varepsilon_{xt}-\varepsilon_{x0})$ and $\log\left(\frac{t}{t_{0}}\right)$ corresponds to a straight line with an intercept at m and slope of n. The derived values of the power law parameters m  and n are listed in Table 3.

The results obtained from the static tests for both types of samples are treated independently for ease of analytical modelling for deriving creep parameters and creep amplitude, as well as evaluating the adaptation of stress-independent and stress-dependent variables. It was determined that the creep amplitude of the FT samples shows greater variation than that of the RT samples.

Regarding the creep parameters, m  is a stress-dependent parameter that varies with different stress levels and applied conditions. According to Findley’s power law, the parameter n is considered as a stress-independent material constant. The material constant n exhibits a slight variation between the RT, FT, and FT-L samples. This variation can be attributed to the fact that n is a temperature- and humidity-dependent parameter. Another possible cause is the different creep behaviors of HFRP bars under different environmental conditions observed in this study. During freeze–thaw treatment, the presence of residual stress in the HFRP bars or induced thermal stress during thermal cycling affects the resin matrix and matrix/fiber interface. Stress becomes more severe in the presence of moisture and sustained loads during freeze–thaw treatment. On the basis of our results, the increase in creep strain and decrease in short-term properties of the FT samples can be attributed to the moisture uptake, matrix softening, and micro-defects that occurred in the resin matrix and matrix/fiber interface during freeze–thaw treatment. This is one potential reason for the variation in the material parameter n. Table 4 compares the average values of creep parameters obtained by other researchers. There is no specific proof to show the correlation between material constants, material types, and loading types.

### 3.4. Prediction of Viscoelastic Properties

Considering Findley’s power law in Equation (1), for the creep of HFRP bars with various stress levels, the constants $\varepsilon_{x0}$ and m can be expressed as hyperbolic functions of applied stress as follows:

(2) $$\varepsilon_{x0}=\varepsilon \prime_{x0}\;\sinh(\frac{\sigma_{x0}}{\sigma_{x\varepsilon }}),\;m=m^{\prime }\sinh(\frac{\sigma_{x0}}{\sigma_{xm}})$$

In these hyperbolic equations, the variables $\varepsilon \prime_{x0}$, $\sigma_{x\varepsilon }$, $m^{\prime }$, and $\sigma_{xm}$. are constants derived from the creep experiments. These constants and n are all independent of the stress levels utilized in our experiments. Substituting Equation (2) into Equation (1) yields:

(3) $$\varepsilon_{xt}=\varepsilon \prime_{x0}\sinh(\frac{\sigma_{x0}}{\sigma_{x\varepsilon }})+m\prime \left(\frac{t}{t_{0}}{)}^{n}\sinh(\frac{\sigma_{x0}}{\sigma_{xm}}\right)$$

**Figure 10:**
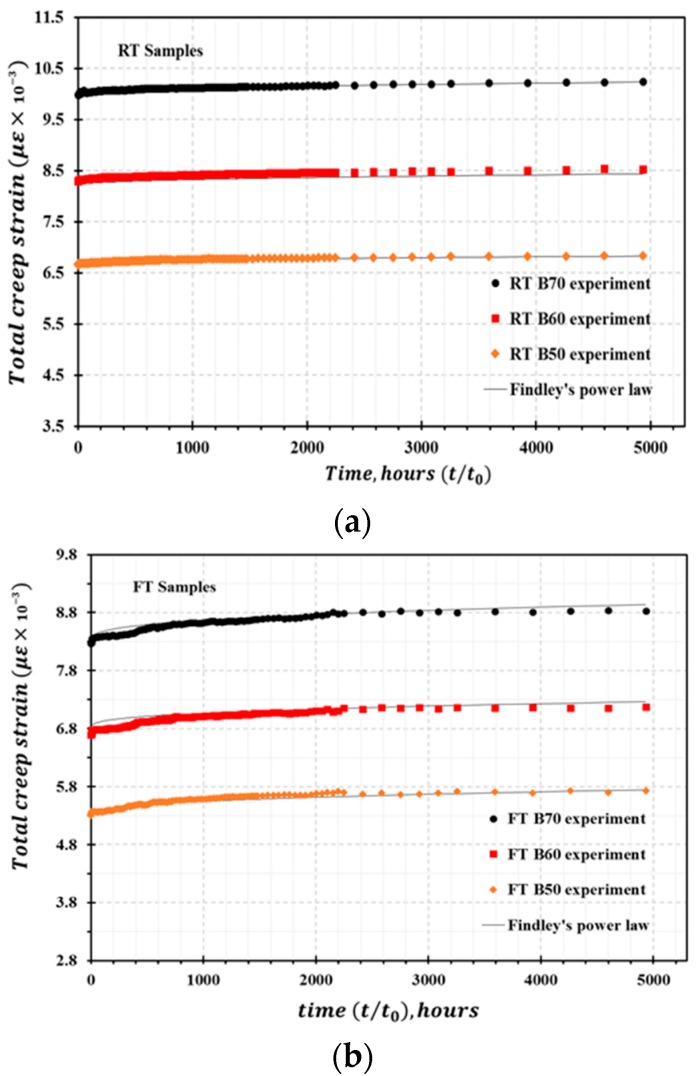

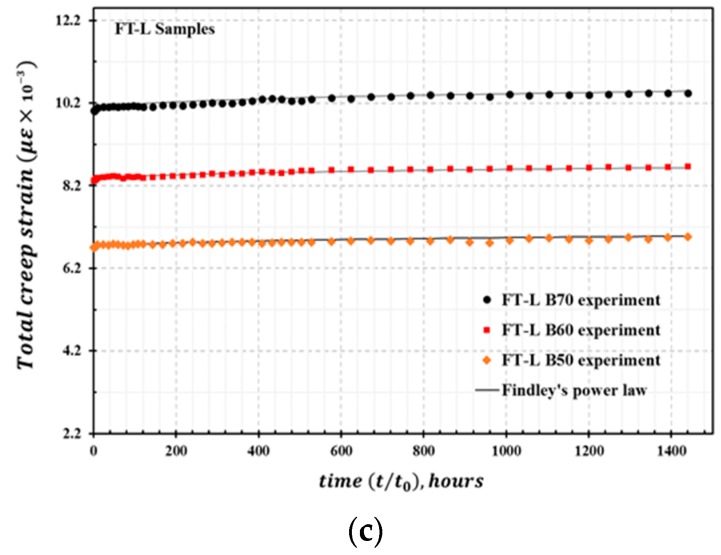
Total creep strain curve derived from theoretical prediction compared with experimental strain: (**a**) RT samples, (**b**) FT samples, and (**c**) FT-L samples.

**Figure 11:**
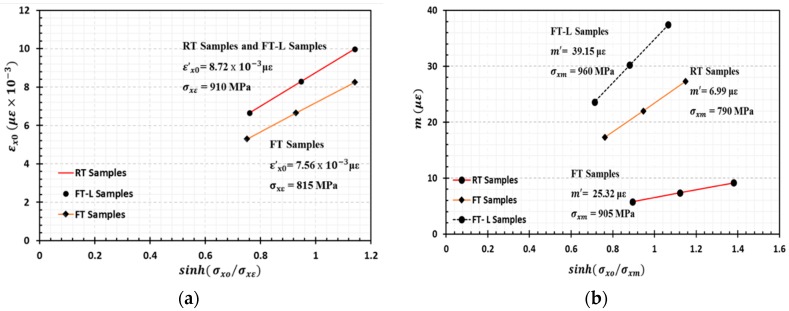
Evaluation of creep parameters (**a**) $\varepsilon \prime_{x0}\;$ and $\sigma_{x\varepsilon }$, (**b**) $\sigma_{xm}$ and m′.

Equation (3) is utilized for predicting the time-dependent viscoelastic strain of HFRP bars. To determine the constants in the transient and instantaneous strain components, Equation (2) was plotted by applying curve fitting methods, where the values of $\sigma_{x\varepsilon }$ and $\sigma_{xm}$ were selected to linearize the curve. $\varepsilon \prime_{x0}\;$ and m′ define the slope of the curve. Figure 10a–c present the total creep strain derived from analytical modelling utilizing Findley’s power law compared with the experimental total creep strain as a function of time for RT, FT, and FT-L samples, respectively. The linear relationships between $\varepsilon_{x0}$ and $\;\sinh(\frac{\sigma_{x0}}{\sigma_{x\varepsilon }})$, and m and $\sinh(\frac{\sigma_{x0}}{\sigma_{xm}})$ are plotted in Figure 11a,b, respectively. The instantaneous strain $\varepsilon \prime_{x0}$ corresponding to the reference stress $\sigma_{x\varepsilon }$ has the same value for the RT and FT-L samples because the stress levels for the creep study of both samples were derived from the same static bending test.

### 3.5. Prediction of Time-Dependent Modulus

On rearranging Equation (3) by applying the Taylor series expansion method, the hyperbolic functions of applied stress can be estimated. By keeping only the linear terms (omitting the cubic and higher-order terms), Equation (3) can be expressed as:

(4) $$\varepsilon_{xt}=\varepsilon \prime_{x0}\left(\frac{\sigma_{x0}}{\sigma_{x\varepsilon }}\right)+m\prime {\left(\frac{t}{t_{0}}\right)}^{n}\left(\frac{\sigma_{x0}}{\sigma_{xm}}\right)$$

Rearranging Equation (4) yields:

(5) $$\varepsilon_{xt}=\sigma_{x0}\left[\left(\frac{\varepsilon \prime_{x0}}{\sigma_{x\varepsilon }}\right)+\left(\frac{m^{\prime }}{\sigma_{xm}}\right)\;\times \;{\left(\frac{t}{t_{0}}\right)}^{n}\right]$$

where,

(6) $$E_{x0}=\;\frac{\sigma_{x\varepsilon }}{\varepsilon_{x0}^{\prime }},\;E_{xt}=\;\frac{\sigma_{xm}}{m^{\prime }}$$

where $E_{x0}$ is the time-independent elastic modulus and $E_{xt}$ is the time-dependent viscoelastic creep modulus.

Substituting Equation (6) in to Equation (5) yields:

(7) $$\varepsilon_{xt}=\sigma_{x0}\left[\left(\frac{1}{E_{x0}}\right)+\left(\frac{1}{E_{xt}}\right)\;\times \;t^{n}\right]$$

(8) $$\varepsilon_{xt}=\;\frac{\sigma_{x0}}{\;E_{x}\;\left(t\right)},\;\;E_{x}\;\left(t\right)=\;\frac{E_{x0}\;\times \;E_{xt}}{E_{xt}+\;E_{x0}\;\times \;t^{n}}$$

Equation (8) can be utilized to determine the time-dependent reduction of the elastic modulus $\left(E_{x0}=68\;\mathrm{GPa}\right)$. The calculated viscoelastic modulus for the RT samples is $E_{xt}=113,018\;\mathrm{GPa}$, for the FT samples is $E_{xt}=35,742\;\mathrm{GPa}$, and for the FT-L samples is $E_{xt}=24,521\;\mathrm{GPa}$. The value of $E_{x0\;}$ is considered as the initial modulus for all samples.

The reduction in the flexural modulus after 25 y can be calculated as follows:

$$E_{x}\;\left(25\;\mathrm{years}\right)=\;\frac{68\times 113,018}{113,018+\;68\times 25{\mathrm{years}}^{0.38}}=63.64\;\mathrm{GPa}\;\mathrm{for}\;\mathrm{RT}\;\mathrm{samples},$$

$$E_{x}\;\left(25\;\mathrm{years}\right)=\;\frac{68\times 35,742}{35,742+\;68\times 25{\mathrm{years}}^{0.37}}=\;57.27\;\mathrm{GPa}\;\mathrm{for}\;\mathrm{FT}\;\mathrm{samples},$$

$$E_{x}\;\left(25\;\mathrm{years}\right)=\;\frac{68\times 24,521}{24,521+\;68\times 25{\mathrm{years}}^{0.35}}=\;56.43\;\mathrm{GPa}\;\mathrm{for}\;\mathrm{FT-L}\;\mathrm{samples}$$

### 3.6. Design Formulation and Verification for Viscoelastic Modulus

To evaluate the time-dependent viscoelastic modulus and determine the accuracy $E_{xt}$ for the HFRP bars, we can apply the long-term design equation proposed by Scott and Zureick [44]. First, we rewrite Equation (8) as:

(9) $$E_{x}\;\left(t\right)=\;\frac{E_{x0\;}}{1+\;\frac{E_{x0}}{E_{xt}}\;\times \;t^{n}}$$

Equation (9) can be rewritten as follows:

(10) $$E_{x}\;\left(t\right)=\;E_{x0}\;\times \;\chi \;\left(t\right)$$

where χ(t) is a time-dependent reduction factor.

(11) $$\chi \;\left(t\right)={\left(1+\frac{Ex_{0}}{E_{xt}}\;\times \;t^{n}\right)}^{-1}$$

(12) $$\chi \;\left(t\right)={\left(1+\frac{1}{\beta }\;\times \;t^{n}\right)}^{-1}$$

where β is the ratio of the viscoelastic modulus to the initial modulus.

Equation (12), can also be expressed as follows:

(13) $$\chi \;\left(t\right)={\left(1+\phi \left(t\right)\right)}^{-1}$$

where

(14) $$\phi \;\left(t\right)=\frac{1}{\beta }\;\times \;t^{n}$$

where ϕ(t) is the time-dependent coefficient of viscosity based on deformation. Table 5 lists the prediction results for the elastic modulus $E_{xt}$, reduction factor χ (t), and coefficient of viscosity  ϕ(t) for RT, FT, and FT-L samples. It should be mentioned that Equation (14) is incorporated in Technical Recommendation of Italian National Research Council [46].

### 3.7. Prediction of Long-Term Flexural Mid-Span Deflection

Findley’s power law in Equation (1) was successfully applied to the modelling of time-dependent viscoelastic properties of HFRP bars. It is also feasible to extend this model to estimate time-dependent creep deflection $\delta_{xt}$. The Findley’s power law expression for $\delta_{xt}$ is defined as:

(15) $$\delta_{xt}=\delta_{x0}+m_{d}{\left(\frac{t}{t_{0}}\right)}^{n_{d}}$$

where $\delta_{x0}$ is the stress-dependent initial deflection and $m_{d}$ and $n_{d}$ are the stress-dependent and stress-independent parameters, respectively, determined by fitting experimental data, similar to the values of m and n for viscoelastic strain evaluation. Equation (15) can be utilized for fitting the experimental creep deflection results of HFRP samples subjected to each stress level in our experiments on RT and FT samples.

To improve the accuracy of prediction and analyze the time-dependent creep deflection of HFRP bars, we can combine Findley’s power law with Euler Bernoulli’s theory [33].

Euler’s mid-span deflection for three-point bending coupled with a viscoelastic modulus $E_{xt}$ is given by the following:

(16) $$\delta_{xt}=\frac{1}{48}\frac{PL^{3}}{IE_{xt}}=\;\delta_{xt}=\frac{1}{48}\frac{PL^{3}}{I}\left[\left(\frac{1}{E_{x0}}\right)+\left(\frac{1}{E_{xt}}\right)\;\times \;t^{n}\right]$$

(17) $$\delta_{xt}=\left[\frac{1}{48}\frac{PL^{3}}{E_{x0}I}\right]+\;\left[\frac{1}{48}\frac{PL^{3}}{E_{xt}I}\right]t^{n}$$

Equation (16) is the simple form of the derived equation, where P represents the load applied, L represents the test span, and I represents the second moment of area. In accordance with the power law, the above Equation (17) can be considered as an explicit function of empirical stress $\sigma_{x0}$ and time t by taking the time-dependent and time-independent components of mid-span deflection and considering the equality of the material constant n.

For HFRP bars in a three-point bending configuration, the equation for predicting time-dependent creep deflection can be expressed as follows:

(18) $$\delta_{xt}^{3p}=\;\sigma_{xt}\left(\frac{L^{2}}{6D}\right)\left[\left(\frac{1}{E_{x0}}\right)+\left(\frac{1}{E_{xt}}\right)\;\times \;t^{n}\right]$$

Equation (18) can be utilized to predict time-dependent creep deflection as follows:

$$\delta_{xt}^{3p}RT=\;\sigma_{xt}\left(\frac{L^{2}}{6D}\right)\left[\left(\frac{1}{68}\right)+\left(\frac{1}{113,018\;}\right)\;\times \;t^{0.38}\right]$$

$$\delta_{xt}^{3p}FT=\;\sigma_{xt}\left(\frac{L^{2}}{6D}\right)\left[\left(\frac{1}{68}\right)+\left(\frac{1}{35,742\;}\right)\;\times \;t^{0.38}\right]$$

where $\sigma_{xt}$ is the empirical stress, L is the test span, and *D* is the diameter of the material. On the basis of the linearization hypothesis, the viscoelastic modulus $E_{xt}$ and short-term modulus $E_{x0}$ are applied as viscoelastic constants.

**Figure 12:**
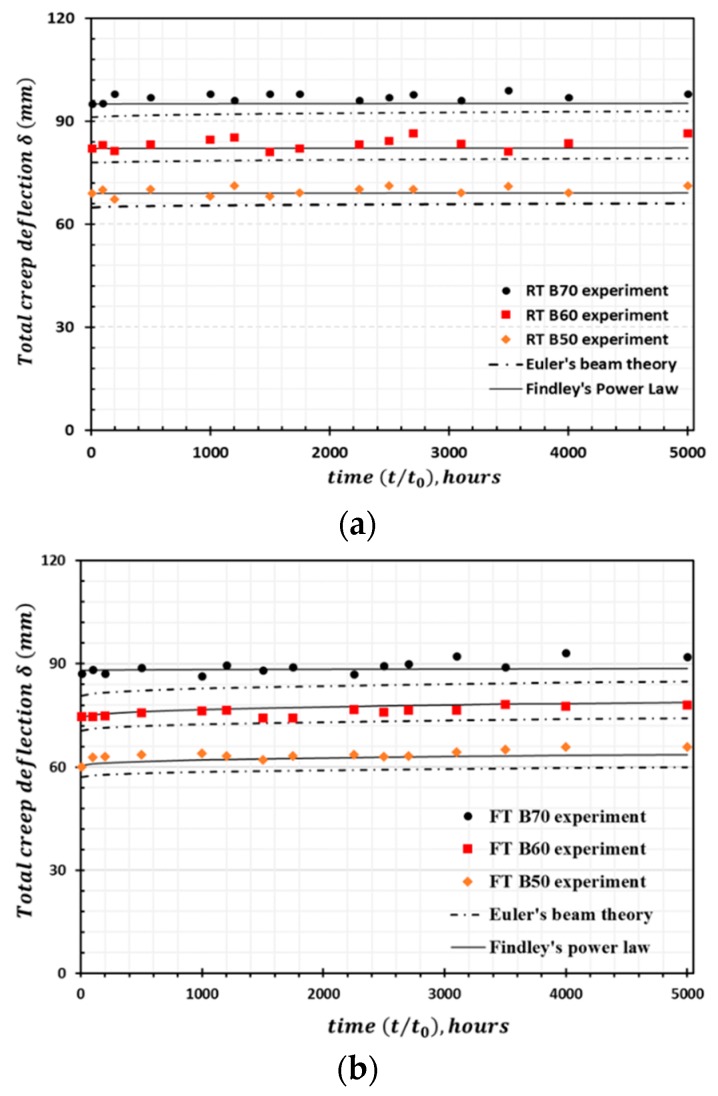
Deflection creep curve (**a**) RT samples and (**b**) FT samples; the plot compares creep deflection derived from the experiment, Findley’s power law model, and Euler Bernoulli’s beam theory results.

On the basis of the prediction results obtained, it was determined that the percentage increase in the deflection of RT samples is less than that of FT samples. Figure 12 presents the total time-dependent creep deflection of RT and FT samples with comparisons between theoretical and experimental deflection. The results predicted by Euler’s beam theory exhibit lesser deflection compared with the actual experimental deflection. In contrast, the deflection predicted utilizing Findley’s power law model is in very good agreement with the experimental data when considering total creep deflection.

Euler’s beam equation for the creep deflection of HFRP samples was designed based on the linear approximation of applied stress utilized in our creep experiments. In our case studies, moderate-to-high stress levels induced large deflection of HFRP bars. Shear deformation and the transition between linear and nonlinear regimes during the long-term loading of HFRP samples also influence the deflection that occurs at higher stress levels. These factors are very difficult to distinguish experimentally and are not considered in Euler’s beam equation. It should be noted that the differences between the predicted initial deflections $\left(\delta_{x0}\right)$ from Euler’s beam equation and the experimental values $\delta_{x0}$ are relatively small. For the RT samples at 50%, 60%, and 70% of the ultimate stress, the deviations in $\delta_{x0}\;$ were 2.21%, 2.29%, and 3.18%, respectively. For the FT samples at 50%, 60%, and 70% of the ultimate stress, the deviations in $\delta_{x0}$ were 2.69%, 4.8%, and 5.6%, respectively.

The initial creep deflection and percentage increase in deflection over 50 y were predicted utilizing Findley’s power law and Euler’s beam theory. Table 6 lists the prediction results. For the highest load level of $\sigma_{x0}/\sigma_{xl}=0.7$, the increase in deflection for t=50  y is in the range of 26.6–30.7% for the FT samples and 11.1–15% for RT samples. The predicted results for the applied climatic conditions indicate that it is unsafe to apply this material above the stress level corresponding to a deflection of $\sigma_{x0}/\sigma_{xl}$ = 0.6.

### 3.8. Microstructural Analysis

**Figure 13:**
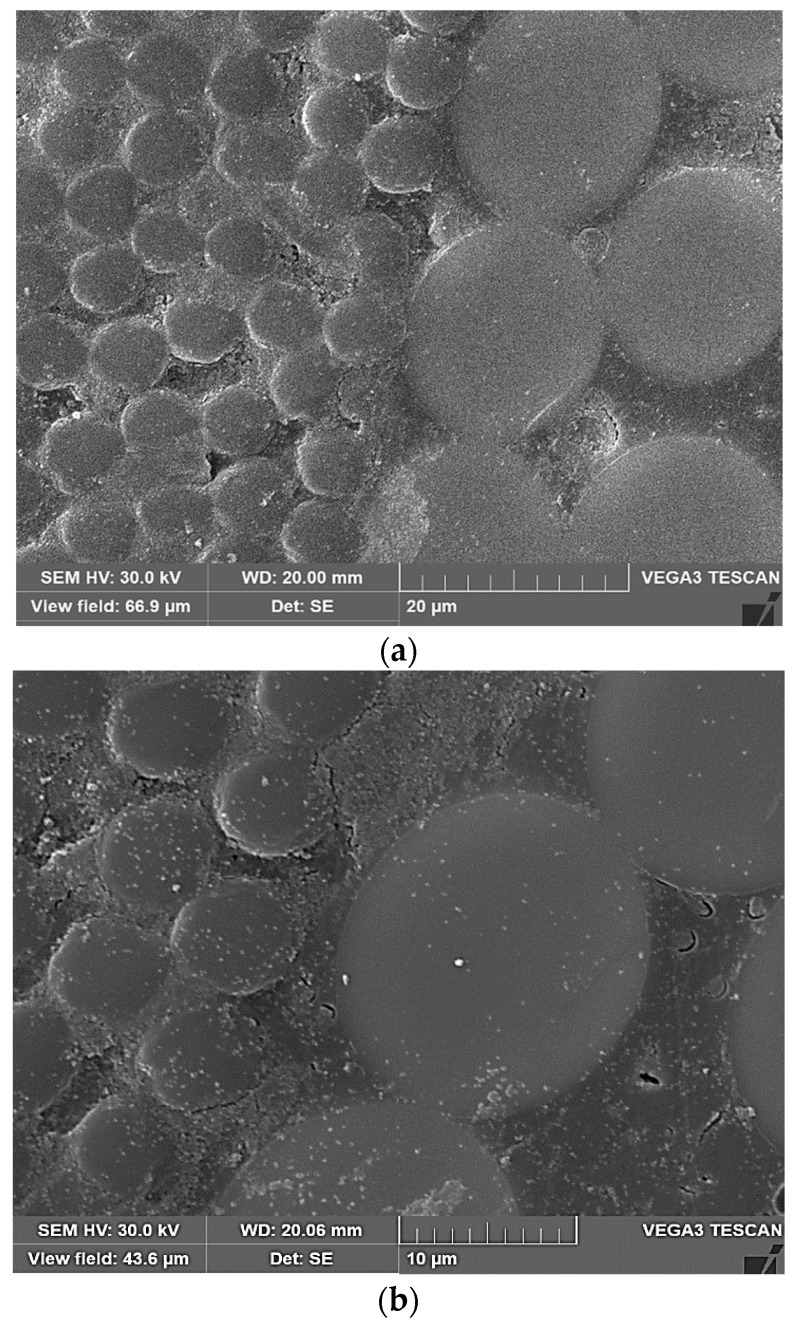
Scanning electron microscopy (SEM) images of HFRP bar specimens: (**a)** Micro-defects in FT Samples; (**b**) Micro-cracks in FT-L samples.

To explain the mechanisms of viscoelastic deformation in HFRP bars during freeze–thaw conditioning for water soaked bars and sustained loading bars in a moist environment, SEM analysis of the bars after our creep study was conducted. Figure 13 presents SEM images of the cross sections of the HFRP bars. Microstructural analysis of the glass/carbon (C/G) hybrid fiber interface was performed in this study because the C/G interface is sensitive to the long-term behavior of this type HFRP bar [2]. A cross section of a water-soaked FT sample is presented in Figure 13a.

It can be seen that the C/G interface suffered from micro-damage and localized degradation, particularly in the area of the carbon fiber/matrix interface. Weak interfacial adhesion between the carbon fibers and resin makes the interface susceptible to thermal stresses. Accelerated degradation is caused by moisture intake during the freeze–thaw treatment of HFRP bars in water. Overall, the relative degree of thermal stress is greater in the presence of moisture. Possible causes for moisture intake include thermal incompatibility between the carbon, glass, and matrix materials, and micro-cracks caused by continuous exposure to thermal cycles. The surface cracking of HFRP bars may occur based on the poor performance of glass fiber shells during water-soaked freeze–thaw cycles caused by pitching, etching, and cracking of the glass fiber shell surface [28]. Such damage makes it easier for moisture to reach the core/shell interface. In our study, the FT-L samples were more susceptible to creep than the FT samples and RT samples. This could be a result of the effects of mechanical loading and induced thermal cycles. Figure 13b presents micro-cracks at the fiber/matrix interface of an FT-L sample under combined loading and thermal cycling in an environment with 75% moisture. Micro-cracks appear in the resin matrix as a result of the residual stress developed by thermal incompatibility and shrinkage of the polymer matrix in HFRP bars during matrix curing. The induced thermal stress during thermal cycles, presence of residual stress, and applied mechanical stress can act together to initiate cracking at the fiber/matrix interface [19].

**Figure 14:**
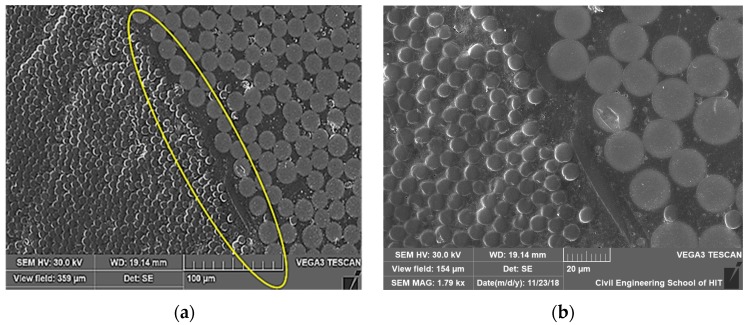
Untreated samples with random presence of unusual gap with a matrix rich area in carbon fiber core/glass fiber shell, interface zone, (**a**) SEM picture of lower amplification; (**b**) amplified SEM picture of the circled zone.

Figure 14a,b reveal the presence of a matrix-rich area at the interface between the carbon fiber core/glass fiber shell of untreated bars. This problem may have developed because of the mismatching CTEs between components in the HFRP system during matrix curing or manufacturing defects that occurred during the pultrusion process. A lack of reinforcement at the interface zone between the glass fiber shell and carbon fiber core or the polymeric nature of the matrix-filled areas of HFRP bars could lead to viscoelastic creep behavior [36]. The effects of creep should be considered when applying HFRP bars as structural materials when moderate-to-high stress is applied over a long period of time under service environmental conditions.

## 4. Conclusions

This study focused on the flexural creep behaviors of HFRP bars and effects of freeze–thaw cycles on such creep behaviors. Static test results indicated a flexural strength reduction of 11% following freeze–thaw cycling treatment in water. On the basis of a creep study under various stress levels (ranging from 50% to 70% of ultimate stress), the amplitude of creep strain in the secondary creep region was observed to be higher for freeze–thaw pretreated HFRP bars compared with that for combined freeze–thaw/mechanically loaded samples. The untreated HFRP bars exhibited a lower percentage of creep compared with those of both of the conditioned samples. By applying Findley’s power law, the time-dependent strain under various stress levels $\;\sigma_{x0}/\sigma_{xl}\approx 50\%\;\mathrm{to}\;70\%$ was analyzed theoretically. The experimental results obtained exhibited good agreement with the theoretical results. Prediction of the time-dependent viscoelastic modulus over 50 y revealed an 11% reduction for the non-treated samples, 19% reduction for the soaked freeze–thaw pretreated samples, and 21% reduction for the combined freeze–thaw pretreated and mechanically loaded samples in a moist environment. Findley’s power law was applied within the context of Euler–Bernoulli’s beam theory to predict the deflection of non-treated samples. We calculated a creep deflection increase of 26.6% for water-soaked freeze–thaw pretreated samples at 70% of the ultimate stress over a service life of 50 y and near the point of failure. The pretreated samples loaded at 60% and 50% of the ultimate load were comparatively safe at their ultimate deflection. In particular, the value of $\sigma_{x0}/\sigma_{xl}\approx 50$% is far from the failure range. Morphological analysis was conducted to identify any micro-defects that occurred in the HFRP bars during our study and to determine the mechanisms of creep in HFRP bars subjected to various environmental conditions.

## Figures and Tables

**Table 1:** Average test results from static bending test with coefficient of variation (CoV).

Short-Term Properties	RT Samples, (CoV [%])	FT Samples, (CoV [%])
Flexural modulus $E_{x0}$ (GPa)	68, (14.96)	68, (14.96)
Ultimate stress $\sigma_{xl}$ (MPa)	1272, (10.84)	1130, (8.31)
Ultimate strain $\varepsilon_{xl}$ (%)	1.4, (11.29)	1.2, (12.82)
Maximum deflection $\delta_{fl}$ (mm)	136, (6.68)	125, (7.32)
Maximum load $f_{l}$ (KN)	4.15, (8.13)	3.68, (8.25)

**Table 2:** The average values of different stress levels and corresponding strain for each samples used in this creep test.

RT Samples	$\sigma_{x0}\;(\mathrm{MPa})$	$\varepsilon_{x0}$ $\;(\;\mu \varepsilon \times 10^{-3})$
RT B50 ($\sigma_{x0}/\sigma_{xl}\approx 0.5$)	638	6.66
RT B60 ($\sigma_{x0}/\sigma_{xl}\approx 0.6$)	767	8.29
RT B70 ($\sigma_{x0}/\sigma_{xl}\approx 0.7$)	890	9.98
Average CoV (%)	10.84	8.31
FT Samples		
FT B50 ($\sigma_{x0}/\sigma_{xl}\approx 0.5$)	566	5.50
FT B60 ($\sigma_{x0}/\sigma_{xl}\approx 0.6$)	678	6.69
FT B70 ($\sigma_{x0}/\sigma_{xl}\approx 0.7$)	795	8.25
Average CoV (%)	11.29	12.82
FT-L Samples		
FT-L B50 ($\sigma_{x0}/\sigma_{xl}\approx 0.5$)	638	6.66
FT-L B60 ($\sigma_{x0}/\sigma_{xl}\approx 0.6$)	767	8.29
FT-L B70 ($\sigma_{x0}/\sigma_{xl}\approx 0.7$)	890	9.98
Average CoV (%)	10.84	8.31

**Table 3:** Average values of material constant *n*, creep parameter *m*, and correlation coefficient $R^{2}$.

Samples	n	m	$R^{2}$
RT B50	0.38	5.77	0.97
RT B60	0.39	7.43	0.96
RT B70	0.38	9.16	0.99
FT B50	0.37	16.50	0.95
FT B60	0.37	21.40	0.96
FT B70	0.36	27.81	0.91
FT-L B50	0.34	23.61	0.88
FT-L B60	0.35	30.23	0.96
FT-L B70	0.35	37.44	0.91

**Table 4:** Average values for material constant n obtained from different creep studies. GFRP, glass–fiber-reinforced polymer (FRP).

Authors	Creep Loading	Duration (h)	Material (GFRP)	*n*
Bank and Mosallam [31]	flexural	3500	frame	0.33–0.34
McClure and Mohammadi [42]	compression	2500	coupons column	0.25 0.17
Scott and Zureick [43]	compression	10000	coupons	0.23
Choi and Yuan [44]	compression	2500	column 1 column 2	0.15 0.19
Shao and Shanmugam [33]	flexural	9000	sheet piling	0.30–0.36
Mário F. Sá [32]	flexural	1680	coupons web coupons Flange beams	0.21–0.22 0.33–0.36 0.31
Mário F. Sá [35]	flexural	3600	panel	0.19

**Table 5:** Prediction of time dependent flexural modulus $\;E_{xt}$, time-dependent reduction factors χ (t), and coefficient of viscosity ϕ (t).

Time Years	(*E_xt_*) Reduction in Modulus (%)			*χ* (*t*) Reduction Factor			*ϕ* (*t*) Coefficient of Viscosity		
	RT Samples	FT-L Samples	FT Samples	RT Samples	FT-L Samples	FT Samples	RT Samples	FT-L Samples	FT Samples
5	4.31	10.47	8.98	0.94	0.90	0.91	0.065	0.1	0.98
10	5.52	12.97	11.31	0.92	0.87	0.88	0.086	0.14	0.13
25	7.38	17.02	15.18	0.89	0.82	0.84	0.1	0.20	0.17
40	9.61	19.48	17.64	0.87	0.80	0.81	0.13	0.24	0.21
50	10.75	21.00	19.00	0.86	0.79	0.80	0.15	0.26	0.23

**Table 6:** Prediction results of time-dependent deflection of RT and FT samples.

YEARS	RT B70	FT B70	RT B60	FT B60	RT B50	FT B50
	[Percentage Increase in Creep Deflection (%), (*Findley’s Power Law/Euler’s Beam Theory*)]					
5	6.02/5.83	13.9/11.2	4.8/4.2	10.8/10.4	4.84/3.41	10.9/10.04
10	8.2/6.4	17.5/14.4	6.3/5.8	14.01/13.2	6.3/4.81	13.4/12.1
25	11.08/9.03	25.8/21.2	9.1/7.8	20.1/18.5	8.94/7.01	19.1/17.4
40	13.21/9.45	28.9/25.1	10.7/9.2	24.7/22.1	10.5/8.69	23.6/20.9
50	15/11.1	30.7/26.6	11.7/10.2	25.6/23.9	11.14/9.4	24.4/21.6
